# Lifestyle and prevalence of dysmenorrhea among Spanish female university students

**DOI:** 10.1371/journal.pone.0201894

**Published:** 2018-08-10

**Authors:** Elia Fernández-Martínez, María Dolores Onieva-Zafra, María Laura Parra-Fernández

**Affiliations:** Department of Nursing, Physiotherapy and Occupational Therapy, Faculty of Nursing, University of Castilla-La Mancha, Ciudad Real, Spain; Universidad Miguel Hernandez de Elche, SPAIN

## Abstract

The aim of this study was to determine the prevalence of primary dysmenorrhea in a sample of Spanish university students, and to describe their menstrual characteristics, lifestyle habits and associated risk factors. This cross-sectional study was conducted with a total of 258 young female university students recruited from the Ciudad Real Faculty of Nursing, with a mean age of 20.63± 3.32 years. An anonymous self-report questionnaire was used to collect data from students. This included sociodemographic characteristics, lifestyle habits, gynecological personal history and the severity of pain using the visual analogue scale. The statistical analysis of the data included calculation of the mean, percentages, chi-square analysis of the data and logistic regression. The prevalence of dysmenorrhea was of 74.8% (n = 193) with a mean pain severity of 6.88 (±1.71). Our results show that 38.3% of students described their menstrual pain as severe and 58% as moderate. The bivariate analysis showed statistically significant differences between students with and without dysmenorrhea: a higher proportion of women with dysmenorrhea had a greater duration of the menstruation flow (*p* = .003), a longer duration of the menstrual cycle (*p* = .046), were not using the oral contraceptive pill (*p* = .026) and had a family history of dysmenorrhea (*p* = .001). Backward step-wise binary logistic regression analysis using all the significant bivariate variables including lifestyle variables revealed the following risk factors: drinking cola drinks, duration of the menstrual flow, eating meat and having a first-degree relative affected by dysmenorrhea.

## Introduction

Menstruation is a normal physiological process that occurs approximately every month in women and which can lead to a certain level of discomfort and pain, although without this incapacitating or affecting their normal daily activity. In contrast, painful dysmenorrhea or menstruation is a common reason for gynecological consultation among adolescents and women, affecting close to 90% of women of reproductive age [1–3]. Dysmenorrhea can be classified as primary, when the referred pain is not related with a prior pelvic illness and is associated with a normal ovulatory cycle, or secondary, when there are signs of pelvic pathology [4].

In primary dysmenorrhea, the patient refers a low abdominal pain that is moderate to intense and is usually accompanied by symptoms such as nausea, tiredness, irritability, headache, dizziness, vomiting and diarrhea. The symptoms may occur before and/or during menstruation, and last up to three days after menstruation, decreasing in intensity from the first day [5]. Among the risk factors related with the episodes of primary dysmenorrhea and identified in the literature, are the appearance of menarche at an early age, a family history of dysmenorrhea, long menstrual cycles, cigarette smoking, alcohol and caffeine consumption, poor sleep hygiene, certain dietary habits, lack of exercise, obesity and having a stressful lifestyle[5–7].

The worldwide prevalence of dysmenorrhea varies considerably among countries, ranging between 50% and 90%[8–11]. This is partly due to the definition of dysmenorrhea itself and/or the way this is measured [1–3]. In a study of Turkish university students, the prevalence was of 87.7%[12], compared to 85.4% in Ethiopia [13], 88% in young Australians [9], 89.9% in university students in Iran [10] and 64% in a sample of Mexican university students[11].

The morbidity of dysmenorrhea has a significant impact on public health as it is one of the first causes of school and work absenteeism, and can therefore lead to higher health costs and reduced work/academic effectiveness, plus a major decrease to the quality of life of those affected[12]. Despite the high prevalence, many studies have reported that women who suffer from dysmenorrhea do not seek medical care and/or consult with other health professionals, and are unaware of how to apply alternative therapy[14]. Until now, few studies have examined the prevalence of dysmenorrhea among samples of university students in Spain[15]. This is important in order to further our understanding of this issue and to research the risk factors that have been extensively studied in other populations.

The aim of this study was to determine the prevalence of primary dysmenorrhea among Spanish university students, and to describe their menstrual characteristics, lifestyle habits and associated risk factors.

## Material and methods

### Study design, setting and participants

This descriptive study was carried out with female students who were enrolled at the Ciudad Real Nursing Faculty in the University of Castilla-La-Mancha between May and June of 2017. A prior sample size calculation was not performed, rather the sample size was based on the entire population of female students, as described below. Retrospectively, we verified that the sample size was large enough to estimate the prevalence of dysmenorrhea in the study population, considering a precision of 3% and a type I error of 5%. And the sample error with our sample was 3%. The eligibility criteria for this study included female students enrolled in an academic year of a degree course taught at the university, and who were present in the classroom when the researcher visited to collect data. Furthermore, all participants had to provide informed consent prior to participation. The population of female students studying at the university was 340 students. In total, 258(76%) agreed to participate in this study.

### Data collection instruments

Data were collected from the female students in the study group using a self-report questionnaire which was specifically designed for this study after researching previous dysmenorrhea studies available[1,10,12,16]. The questionnaire was anonymously completed by each participant. Completion of the questionnaire took between 10 to 15 minutes, during which one of the researchers was present in the classroom. The self-report questionnaire was structured in two parts. The first part included 26 questions regarding the students’ sociodemographic characteristics and lifestyle habits. The second part featured 23 questions regarding the students’ gynecological personal history. Women who reported menstruation pain had to complete additional questions regarding the characteristics of their pain and the influence the same had on their ability to perform everyday activities. To measure pain intensity, a 10-cm horizontal visual analog scale (VAS) was used. The descriptors 0 (no pain) on the left and 10 (worst pain) on the right were used. Scores from the VAS were categorized as mild, moderate and severe based on previous literature [17,18]. A score of 1–3 was considered mild pain, 4–6 was considered moderate pain and 7–10 was considered severe pain.[9,19].

### Data analysis

Data analyses was carried out using the Statistical Software Package for the Social Sciences (SPSS), version 23. Data were examined via the mean, standard deviation for quantitative variables and the frequency and percentage for qualitative variables. The Pearson chi-square, the exact Fisher test, the U Mann Whitney Test, the Kruskal-Wallis and the Student’s T-test and binary logistic regression (backward stepwise were employed for the statistical analysis. The significance level was established at p < 0.05 with a corresponding 95% confidence interval.

### Ethical considerations

The study protocol was approved by the Clinical Research Ethics Committee at the Ciudad-Real General Hospital (approval number C-105). All procedures were followed in accordance with the Helsinki Declaration. Permission to conduct the study was obtained from the management of the Nursing Faculty. Before data were collected, all students were informed of the purpose of the study and written informed consent was obtained. In addition, all participants were assured that their anonymity and confidentiality would be maintained and that they were entitled to drop out of the study at any time.

## Results

The mean age of participants was 20.63± 3.32 years (range 18–45 years) and the mean age of menarche was 12.49 ±1.45 years (range 8–17 years). None of the students were pregnant and only three students had children. Additional characteristics of the participants are shown in Table 1. Around 80% of the students who suffered from dysmenorrhea had a menstrual flow that lasted over five days, with a menstrual cycle frequency of over 29 days duration (79.9%). The bivariate analysis showed statistically significant differences between students with dysmenorrhea and those without, regarding the following variables: duration of the menstruation flow (p < .003), frequency of the menstrual cycle (p < .048), oral contraceptive pill (*p* < .026) and having a family history of dysmenorrhea (*p* < .001).

**Table 1:** Socio-demographic characteristics of female participants in the study group.

	DYSMENORRHEA				
		YES 193 (74.8%)	NO 65 (25.2%)	TOTAL 258 (100%)	p value
**Age**	≤ 20 years	122(73.9%)	43(26.1%)	165(64%)	p = .679
	21–24 years	60(77.9%)	17(22.1%)	77(29.8%)	
	≥ 25 years	11(68.8%)	5(31.3%)	16(6.2%)	
**BMI**	<18,5	25(80.6%)	6(19.4%)	31(12%)	p = .725
	18,5–24,99	143(74.1%)	50(25.9%)	193(74.8%)	
	≥ 25	25(73.5%)	9(26.5%)	34(13.2%)	
**Living context**	Rural	35(79.5%)	9(20.5%)	44(17.1%)	p = .427
	Urban	158(73.8%)	56(26.2%)	214(82.9%)	
**Regularity of cycle**	Irregular	64(79%)	17(21%)	81(31.4%)	p = .292
	Regular	129(72.9%)	48(27.1%)	177(68.6%)	
**Age of menarche**	≤12 year	104(71.7%)	41(28.3%)	145(56.2%)	p = .344
	13–14 years	68(77.3%)	20(22.7%)	88(34.1%)	
	≥15 year	21(84%)	4(16%)	25(9.7%)	
**N° days spotting**	3 to 5	119(69.2%)	53(30.8%)	172(66.7%)	p = .003*
	6 to 11	74(86%)	12(14%)	86(33.3%)	
**Cycle frequency**	≤ 21 days	2(40%)	3(60%)	5(1.9%)	p = .046*
	22 to 28 days	84(70.6%)	35(29.4%)	119(46.1%)	
	≥29 days	107(79.9%)	27(20.1%)	134(51.9%)	
**Family member with dysmenorrhea**	Yes	137(81.1%)	32(18.9%)	169(65.5%)	p = .001*
	No	56(62.8%)	33(37.1%)	89(34.5%)	

*p < .05

The prevalence of dysmenorrhea in the study group was 74.8% (n = 193) (95% CI = 69.47%-80.14%). The dysmenorrhea characteristics of the students are presented in Table 2. The mean severity of pain was 6.88 (±1.71) on the VAS. Of all students, 38.3% described their menstrual pain as severe and 58% as moderate. In addition to the menstrual pain, edema (92.7%), irritability (81.9%) and fatigue (79.3%) were the most commonly reported symptoms. Moreover, of the sample experiencing dysmenorrhea, 75.6% of the students reported that their daily activities were affected. In total, 91.2% of students with dysmenorrhea were taking analgesics, of which 77.7% mainly self-medicated when they suffered from a worsening of symptoms.

**Table 2:** Dysmenorrhea characteristics of the students.

		VAS FOR PAIN				
		Mild	Moderate	Severe	Total	p value
**Cycle**	Regular	6(4.7%)	75(58.1%)	48(37.2%)	129(68.8%)	p = .316
	Irregular	0(0%)	37(57.8%)	27(42.2%)	64(33.2%)	
**Cycle duration**	Equal or less than 21 days	0(0%)	1(50%)	1(50%)	2 (1%)	p = .907
	22 to 28 days	3 (3.6%)	48(57.1%)	33(39.3%)	84(43.5%)	
	29 days or more	3(2.8%)	63(58.9%)	41(38.3%)	107(55.4%)	
**N° days spotting**	≤ 5 days	3(2.5%)	72(60.5%)	44(37%)	119(61.7%)	p = .605
	≥6 days	3(4.1%)	40(54.1%)	31(41.9%)	74(38.3%)	
**Moment at which pain is perceived**	2–3 days before menstruation	0(0%)	36(59%)	25(41%)	61(31.6%)	p = .746
	1^st^ day of menstruation	6(5.4%)	62(55.9%)	43(38.7%)	111(57.5%)	
	Second or third day	0(0%)	14(66.7%)	7(33%)	21(10.9%)	
**Uses analgesics**	No	1(5.9%)	13(76.5%)	3(17.6%)	17(8.8%)	p = .022*
	Yes, self-medicates	5(3.3%)	88(58.7%)	57(38%)	150(77.7%)	
	Yes, with a prescription	0(%)	11(42.3%)	15(57.7%)	26(13.5%)	
**When are analgesics taken?** **(multiple response)**	Days prior to menstruation	0(0%)	7(43.8%)	9(56.3%)	16(8.8%)	p = .172
	During menstruation	1(1.6%)	33(53.2%)	28(45.2%)	62(34.1%)	
	When symptoms worsen	3(2.9%)	64(61.5%)	37(35.6%)	104(57.1%)	
**Dizziness**	Yes	0 (0%)	24 (39.3%)	37 (60,7%)	61(31.6%)	p = .000*
	No	6(4.5%)	88(66.7%)	38(28.8%)	132(68.4%)	
**Nausea and vomiting**	Yes	0(0%)	26(40%)	39(60%)	65(33.7%)	p = .000*
	No	6(4.7%)	86(67.2%)	36(28.1%)	128(66.3%)	
**Diarrhea**	Yes	3(3.2%)	54(57.4%)	37(39.4%)	94(48.7%)	p = .906
	No	3(3%)	58(58.6%)	38(38.4%)	99(51.3%)	
**Sleep disorders**	Yes	1(1.7%)	27(45%)	32(53.3%)	60(31.1%)	p = .005*
	No	5(3.8%)	85(63.9%)	43(32.2%)	133(68.9%)	
**Fatigue**	Yes	4(2.6%)	84(54.9%)	65(42.5%)	153(79.3%)	p = .038*
	No	2(5%)	28(70%)	10(25%)	40(20.7%)	
**Swelling**	Yes	5(2.8%)	104(58.1%)	70(39.1%)	179(92.7%)	p = .668
	No	1(7.1%)	8(57.1%)	5(35.7%)	14(7.3%)	
**Headache**	Yes	4(3.9%)	54(52.9%)	44(43.1%)	102(52.8%)	p = .283
	No	2(2.2%)	58(63.7%)	31(34.1%)	91(47.2%)	
**Irritability**	Yes	4(2.5%)	88(55.7%)	66(41.8%)	158(81.9%)	p = .061
	No	2(5.7%)	24(68.6%)	9(25.7%)	35(18.1%)	
**Depressed**	Yes	3(2.5%)	68(55.7%)	51(41.8%)	122(63.2%)	p = .237
	No	3(4.2%)	44(62%)	24(33.8%)	71(36.8%)	
**Effects on daily life**	Yes	1(0.7%)	71(48.6%)	74(50.7%)	146(75.6%)	p = .000*
	No	5(10.6%)	41(87.2%)	1(2.1%)	47(24.4%)	

*p < .05

Table 3 features the characteristics of the study participants regarding lifestyle and dietary habits. In the group with dysmenorrhea, 26% were alcohol consumers compared to 32.31% of the group of students without dysmenorrhea. Regarding cigarette smoking, only 15% of the group with dysmenorrhea smoked, whereas in the group without dysmenorrhea, 23.08% smoked. Among the factors that were statistically significant, there was a statistically significant difference in the consumption of tea, cola drinks, simple sugars, meat and fruit three times a day, and regarding cooking with olive oil.

**Table 3:** Lifestyle characteristics and dietary habits.

Lifestyle and diet		DYSMENORRHEA			p value
		YES 193 (74,8%)	NO 65(25.2%)	TOTAL 258 (100%)	
**Alcohol**	Yes	51(70.8%)	21(29.2%)	72(27.9%)	p = .360
	No	142(76.3%)	44(23.7%)	186(72.1%)	
**Tobacco**	Yes	29(65.9%)	15(34.1%)	44(17.1%)	p = .136
	No	164(76.6%)	50(23.4%)	214(82.9%)	
**Coffee**	Yes	89(74,8%)	30(25,2%)	119(46,4%)	p = .996
	No	104(74,8%)	35(25.2%)	139(53,9%)	
**Tea**	Yes	97(69.8%)	42(30,2%)	139(53.9%)	p = .045*
	No	96(80.7%)	23(19.3%)	119(46,1%)	
**Chocolate**	Yes	161(76.7%)	49(23.3%)	210 (81.4%)	p = .150
	No	32(66.7%)	16(33.3%)	48(18.6%)	
**Energy drinks**	Yes	47(81%)	11(19%)	58(22.5%)	p = .215
	No	146(73%)	54(27%)	200(77.5%)	
**Fast food**	Yes	164(76.6%)	50(23.4%)	214(82.9%)	p = .136
	No	29(65.9%)	15(34.1%)	44(17.1%)	
**Cola drinks**	Yes	130(80.2%)	32(19.8%)	162(62.8%)	p = .009*
	No	63(24.4%)	33(12.8%)	96(37.2%)	
**Simple sugars**	Yes	173(77.2%)	51(22.8%)	224(86.8%)	p = .021*
	No	20(58,8%)	14(41.2%)	34(13.2%)	
**Daily water intake**	< 1liter	41(74.5%)	14(25.5%)	55(21.3%)	p = .874
	1 to 2 liters	127(75.6%)	41(24.4%)	168(65.1%)	
	>2 liters	25(71.4%)	10(28.6%)	35(13.6%)	
**Always cooks with olive oil**	Yes	145(71.4%)	58(28.6%)	203(78.7%	p = .016*
	No	48(87.3%)	7(12.7%)	55(21.3%)	
**Eats fruit daily (minimum 3 pieces of fruit per day)**	Yes	47(65.3%)	25(34.7%)	72(27.9%)	p = .028*
	No	146(78.5%)	40(21.5%)	186(72.1%)	
**Eats dairy daily (minimum 1 per day)**	Yes	148(74.4%)	51(25.6%)	199(77.1%)	p = .786
	No	45(76.3%)	14(23.7%)	59(229%)	
**Whole-grain cereals (at least 1 per week)**	Yes	157(74.4%)	54(25.6%)	211(81.8%)	p = .755
	No	36(76.6%)	11(23.4%)	47(18.2%)	
**Eats meat (compare with vegetarians)**	Yes	192(75,9%)	61(24,1%)	253(98%)	p = .015*
	No	1(20%)	4(80%)	5(1.9%)	
**Fish**	Yes	185(74,9%)	62(25,1%)	247(95,7%)	p = .554
	No	8(72,7%)	3(27,3%)	11(4,3%)	
**Hours of sleep**	< = 6h	131(76.2%)	41(23.8%)	172(66.7%)	p = .593
	From 7 to 8	53(73.6%)	19(26.4%)	72(27.9%)	
	> = 9h	9(64.3%)	5(35.7%)	14(5.4%)	
**Exercise**	Yes	64(69.4%)	28(30.4%)	92(35.7%)	p = .149
	No	129(77.7%)	37(22.3%)	166(64.3%)	
**Oral contraceptives**	Yes	25(61%)	16(39%)	41(15.9%)	p = .026*
	No	168(77.4%)	49(22.6%)	217(84.1%)	

*p < .05

Results of the backward step-wise binary logistic regression analysis using the significant bivariate variables are given in Table 4. The risk of dysmenorrhea in participants who drank cola drinks was 2.19 higher (OR 2.19; 95% CI 1.19–4.04), in those who spotted for over 6 days this was 2.67 (OR 2.67; 95% CI 1.29–5.57) while in those who ate meat this was 20.99 higher (OR 20.99; 95% CI 2.05–215.17) when compared to non-meat eaters. Those with a first level family member with dysmenorrhea presented a risk 2.62 greater than those without (OR 2.62; 95% CI 1.42–4.82). Other risk factors that were not statistically significant predictors in the multivariate model were removed.

**Table 4:** Binary logistic regression analysis of significant variables related to dysmenorrhea.

	OR	95% CI	p values
**Cola drinks**	2.19	1.19–4.04	.012*
**N° days spotting (≥ 6 days)**	2.67	1.29–5.57	.009*
**Meat**	20.99	2.05–215.17	.010*
**1st degree family member**	2.62	1.42–4.82	.002*

*p < .05

Calibration was evaluated using the Hosmer-Lemeshow test (p = .485) and the ROC curve was used to measure discrimination, AUC = 0.70. (95% IC = 0.63–0.73;p<0.01). A cut-off value of 0.69 was estimated with an estimated sensitivity of 65.3% and a specificity of 66.2%.

## Discussion

Dysmenorrhea is an important and common health problem in women of reproductive age that has a negative impact on quality of life. The present study found a prevalence of dysmenorrhea of 74.8%, which is in line with the rates reported in the literature in which the prevalence of dysmenorrhea among university students may vary from 50% to 90% [9,11]. This difference should be cautiously interpreted as it may be due to different selected samples or a different method of defining and measuring dysmenorrhea. In our study, the prevalence of dysmenorrhea was determined according to the presence of menstrual pain in at least three cycles over the last year in a sample of Spanish university students. To the best of our knowledge, in the last 10 years only one study has examined factors associated with increased pain in dysmenorrhea in a sample of Spanish university students in April 2017. [15]

In the present study, regular menstrual cycles were not found to be a significant risk factor for dysmenorrhea. However, the majority of the students who had dysmenorrhea also had regular menstrual cycles (68.8%), as reported in the literature [20]. The cause of the menstrual pain is thought to be the excess amount of prostaglandins released during the regular ovulation cycle, which causes contractions and pain in the uterine tissue[21]. In addition, we were unable to find statically significant increased odds for the occurrence of dysmenorrhea in students who had an earlier menarche. As reported by other studies [22] this is based on the explanation that the odds increase because of the length of time that women are exposed to menses and to prostaglandins. In our study, a large proportion of students with dysmenorrhea (71.7%) reported an early menarche (≤ 12 years old)[23–25]. The small number of students who reported no dysmenorrhea could be why statistical significance was not met in the logistic regression analysis.

According to the bivariate analysis, our study showed that the duration of the menstrual flow, the frequency of the menstrual cycle, taking oral contraceptive pills and having a family history of dysmenorrhea were statistically significant factors, as reported by prior studies [22,26,27]. In the present study, 86% of the students with a longer menstrual flow (over 5 days) and almost 80% of the students with longer cycles (more than 29 days) reported menstrual pain compared to 14% with a longer menstrual flow and 20% with longer cycles who reported no dysmenorrhea. Some researchers have suggested that women with a family history of dysmenorrhea can experience menstrual pain due to behavior learned from their mothers and sisters[28–30]. However, other studies have also suggested a genetic susceptibility to dysmenorrhea[31]. In our study, more than 80% of the students with dysmenorrhea reported having a first-degree family member suffering from pain and discomfort during menses, without specifying the relationship. In future studies, it would be interesting to make the distinction between having an affected mother and/or sisters and the mother’s education as a predictive factor for a daughter’s behavior regarding menstrual pain, as suggested by other authors[32]. Regarding taking the pill, and despite finding significant differences among groups, the later analysis does not include this as a protective factor in the regression equation performed in our study, as reported by prior studies. Most likely, this finding is due to our small sample [5].

In the present study, the mean severity of pain was 6.88 (±1.71) according to the VAS, which was higher than the pain reported in other studies [12,16,33]. Furthermore, 90% of the sample with dysmenorrhea reported severe and moderate pain. A possible explanation for this is the individual’s perception of pain which may be explained by cultural differences and differences in the sample. In the study by Potur et. al. 80% of women with dysmenorrhea of moderate to severe intensity opted for taking analgesics, which is a slightly lower percentage to that found in our study, where 89% of students with moderate and severe pain referred regular use of analgesics. It is likely that this difference could be attributed to a greater perception of pain on behalf of the sample as reflected by the results obtained in the VAS. Regarding self-medication, our results also reflect a slightly higher percentage compared to those provided in the study by Ortiz MI[20] where 67.7% self-medicated themselves. However, we do agree with previous reports regarding the fact that most university students choose self-medication and/or self-care instead of seeking professional advice[11,16,33–35]. The most common co-occurring symptoms were edema, irritability and fatigue, which are similar to those reported in the study by Aktas C. with 34.6% reporting irritability and 21.5% presenting fatigue[36]. These results suggest that dysmenorrhea among young university students must be considered an important health problem in our community, as it conditions and limits daily activities which, in turn, leads to a reduced quality of life and greater absenteeism.

Among the lifestyle factors studied which are related to dysmenorrhea, neither alcohol nor smoking were found to be factors related to a greater risk of suffering from dysmenorrhea, which was also revealed in other studies [5,37–39]. The study by Hong, Jones and Mishra found no significant differences for establishing tobacco smoking as a risk factor for dysmenorrhea. However, in contrast with our study, the prevalence of dysmenorrhea among Australian youth was modestly higher among those who belonged to the group of smokers[5]. In the study by Chen, the prevalence of dysmenorrhea among Chinese youth exposed to environmental tobacco smoke (ETS) was slightly higher when compared to that of women who were not exposed and significant values were only found when the exposure was due to the large number of cigarettes smoked[37]. However, in a study with a smaller sample, Chung found no significance of cigarette smoking, passive exposure to smoke or alcohol consumption in relation to having a greater risk of suffering dysmenorrhea[39]. In our study, both the prevalence of smokers as well as that of alcohol drinkers was less than that reported in other studies involving university students [5]. Furthermore, there was no quantification of the alcohol, cigarettes smoked or the exposure to tobacco smoke.

Indeed, some authors suggest a lack of agreement among the different researchers regarding the identification of the range of risk factors for developing dysmenorrhea[40]. Regarding consumption of olive oil, our findings indicate there is a greater proportion of students with dysmenorrhea among those who do not consume olive oil on a daily basis. Prior studies have demonstrated the anti-inflammatory power of olive oil and fish oil regarding the action of the Omegas 3 and 6 in the production of prostaglandins [41–44]. Nonetheless, an important limitation for the interpretation of this result is the fact that, occasionally, a person may be unaware of the oil used during cooking, for example when they are not the ones preparing the meal. In contrast, drinking cola drinks and eating meat are revealed as being risk factors for suffering from dysmenorrhea. In several studies, vegetarian diets and/or the consumption of fruits and vegetables are related to the decrease of estrogen activity and therefore the decrease of the frequency of dysmenorrhea[29,45,46]. In our study, the consumption of fruit showed significant differences, as suggested by the study by Parazzini et al. In relation to endometriosis, however, this has not been revealed as a predictive factor in the logistic regression[46]. Regarding the consumption of cola drinks, a study performed on a sample of 400 Ethiopian university students also identified cola as a risk factor[13].

The findings from this study should be interpreted in light of limitations. Retrospective reporting of menstrual symptoms may lead to inaccurate reporting of symptoms and the fact that this was assessed by a self-report questionnaire should be considered. Furthermore, in relation to the sample, our study only included women from a single faculty, therefore it would be important to repeat this study in other study populations.

In conclusion, although dysmenorrhea is a common problem among the young adult population, this has been poorly studied to date in the context of Spanish university students. Our findings reveal that dysmenorrhea affects a large part of our university population, and is a problem that impacts students’ daily life. There are known non-modifiable risk factors in the literature, also identified in our study, which increase the probability of suffering dysmenorrhea, such as, for example, having a first-degree family member who suffers the problem. However, concerning lifestyle and eating habits, and based on our results and previous reports, further studies are necessary to provide or confirm recommendations on the most advisable diets or lifestyle habits for reducing the risk of suffering from dysmenorrhea.

## Supporting information

S1 File This is the S1 File. Data.
(XLSX)

S2 File This is the S2 File. Questionnaire.
(DOCX)
