# Supplementary material for: Lifestyle and prevalence of dysmenorrhea among Spanish female university students
Source: PLoS One. 2018 Aug 10;13(8):e0201894. doi: 10.1371/journal.pone.0201894 (PMC6086430; doi:10.1371/journal.pone.0201894)
Supplement: S2 File — Questionnaire. (DOCX) [file pone.0201894.s002.docx]

**QUESTIONNAIRE**

**SPANISH VERSION**

**Cuestionario Sociodemográfico**

1.- Edad (en años)

2.- Estado civil ( civil status)

- Soltera
- Casada
- Divorciada
- Viuda

3.- Peso (kg)

4.- Estatura (m)

**Estilo de vida**

5.- ¿En qué ámbito vive?

- Rural
- Urbano

6.- ¿Realiza ejercicio físico?

- Sí
- No

7.- Indique cuantas horas a la semana realiza ejercicio físico.

0🡪15

8.- ¿Usted fuma?

- Si
- No

9.- Si ha contestado si a la pregunta anterior. ¿Cuantos cigarrillos fuma al día?

10.- Indique el número de horas que duerme al día.

11.- ¿Bebe alcohol?

- Si
- No

12.- ¿Consume alguna droga?

- Si
- No

13.- ¿Consume comida rápida?

- Si
- No

14.- ¿Consume bebidas energéticas?

- Si
- No

15.- ¿Consume bebidas a base de cola?

- Si
- No

16.- ¿Consume té?

- Si
- No

17. ¿Consume café?

- Si, indique el número de tazas al día:
- No

18.- ¿Consume azúcares simples a diario: galletas, bollería, dulces, caramelos?

- Si
- No

19.- ¿Cuánta agua bebe al día?

- Menos de 1 litro
- De 1 a 2 litros
- Más de 2 litros

20.- ¿Consume verdura a diario?

- Si
- No

21.- ¿Consume pescado todas las semanas?

- Si
- No

22.- ¿Consume carne todas las semanas?

- Si
- No

23.- ¿Consume lácteos a diario?

- Si
- No

24.- ¿Cuántas raciones de cereales integrales consume a la semana?

25.- ¿Usa aceite de oliva para cocinar siempre?

- Si
- No

26.- ¿Consume al menos 3 piezas de fruta al día?

- Si
- No

**Antecedentes personales y ginecológicos**

27.- Edad de la menarquia (primera menstruación)

28.- ¿Cuántos días suele manchar durante la menstruación?

29.- ¿Su ciclo menstrual es regular? En relación a sus menstruaciones en los últimos 6 meses

- Si, es regular
- No, es irregular

30.- Indique el número de días que transcurre entre el primer día de su menstruación y el primer día de su siguiente menstruación

31.- ¿Usted tiene dismenorrea? Entendiendo como tal dolor pélvico y/o lumbar antes y/ol durante la menstruación

- Si
- No

32.- ¿Tiene algún familiar de primer grado( su madre y/o hermanas ) dismenorrea?

- Si
- No

33.- En relación con la menstruación, ¿siente mareo?

- Si
- No

34.- En relación con la menstruación, ¿siente hinchazón?

- Si
- No

35.- En relación con la menstruación, ¿siente dolor de cabeza?

- Si
- No

36.- En relación con la menstruación, ¿siente nauseas o vómitos?

- Si
- No

37.- En relación con la menstruación, ¿siente irritabilidad?

- Si
- No

38.- En relación con la menstruación, ¿se siente deprimido?

- Si
- No

39.- En relación con la menstruación, ¿tiene diarreas?

- Si
- No

40.- En relación con la menstruación, ¿se le altera el sueño?

- Si
- No

41.- En relación con la menstruación, ¿percibe fatiga o cansancio?

- Si
- No

42.- Si usted padece dismenorrea: Indique la intensidad de la misma en una escala del 0 a 10, en el uno es un dolor mínimo y el 10 es un máximo dolor (no conteste a esta pregunta si usted no padece dismenorrea)

43.- Indique el día o número de días que percibe dolor en relación a su menstruación.

- 2-3-antes de su menstración
- El primer día de la menstruación
- El segundo o tercer día de menstruación
- No tengo dolor con la menstruación (dismenorrea)

44.- ¿Siente que alguno de estos síntomas: dismenorrea y/o otros síntomas descritos en la pregunta anterior, le limitan en sus actividades diarias ( acudir a clase, acudir a prácticas, concentrarse, realizar deporte…)?

- Si
- No

45.- ¿Tiene usted hijos?

- Sí, por parto vaginal
- Sí, por cesárea
- No
- Otros: ……………….

46.-¿Está usted embarazada?

- Si
- No

47.- ¿Utiliza algún método anticonceptivo hormonal?

- Sí
- No

48.- Usa analgésico para aliviar sus síntomas menstruales.

- No
- Sí, me automedico
- Sí, con prescripción

49. Si toma algún analgésico durante la menstruación ¿ Cuándo los toma?

- Días previos a la menstruación
- Durante la menstruación
- Cuando los síntomas se agravan

**QUESTIONNAIRE**

**ENGLISH VERSION**

**Socio-demographic questionnaire**

1. Age (years)

2. Marital status (civil status)

- Single,
- Married,
- Divorced,
- Widow

3. Weight (Kg)

4. Height (m)

**Lifestyle**

5. Where do you live?

- Rural
- Urban

6. Do you exercise?

- Yes
- No

7. Indicate how many hours per week you do physical exercise.

0…...>15

8. Do you smoke?

- Yes
- No

9. If you answered yes to the question above. How many cigarettes do you smoke per day?

10. Please indicate the number of hours sleep a day.

11. Do you drink alcohol?

- Yes
- No

12. Do you take any drugs?

- Yes
- No

13. Do you consume fast food: hamburger, Pizza, food... ready?

- Yes
- No

14. Do you drink energy drinks?

- Yes
- No

15. Do you drink cola drinks?

- Yes
- No

16. Do you drink tea?

- Yes
- No

17. Do you drink coffee?

- Yes, Please indicate the number of cups of coffee consumed per day
- No

18. Do you eat sugars: cookies, pastries, sweets, candies.. daily?

- Yes
- No

19. How much water do you drink a day?

- Less than 1 liter
- 1 to 2 liters
- More than 2 liters

20. Do you eat vegetable daily?

- Yes
- No

21. Do you eat fish every week?

- Yes
- No

22. Do you eat met every week?

- Yes
- No

23. Do you take dairy daily?

- Yes
- No

24. How many servings of whole grain cereal take a week?

25. Do you always use olive oil for cooking?

- Yes.
- No.

26. Do you eat at lees 3 servings fruits per day?

- Yes
- No

**Personal and gynecological history**

27. Age of menarche (your first menstruation?)

28. How many days do you usually spot during your menstruation ?(in spotting days per cycle)

0-10

29. Is your menstrual cycle regular? In relation to their periods in the last 6 months

- Yes, it is regular
- No, it is regular

30. Indicate the number of days between the first day of your menstruation and the first day of your next period (write the number in days)

31. Do you have dysmenorrhea? Understanding that as a pelvic and/or back pain before and / or during menstruation

- Yes
- No

32. Do you have any first-degree relative (your mother and / or sisters) with dysmenorrhea?

- Yes
- No

33. In relation to menstruation, do you feel dizziness?

- Yes
- No

34. In relation to menstruation, do you feel swelling?

- Yes
- No

35. In relation to menstruation, do you feel headache?

- Yes
- No

36. In connection with menstruation, do you feel nausea or vomiting?

- Yes
- No

37. In relation to menstruation, do you feel irritability?

- Yes
- No

38. In connection with menstruation, do you feel depressed?

- Yes
- No

39. In connection with menstruation, do you have diarrhea?

- Yes
- No

40. In connection with menstruation, is your sleep disturbed?

- Yes
- No

41. In connection with menstruation, do you perceive fatigue or tiredness?

- Yes
- No

42. If you suffer from dysmenorrhea: indicate the intensity on a scale of 0 to 10, in one it is a minimal pain and 10 is the maximum pain (no answer to this question if you do not suffer from dysmenorrhea)

0-10

43. Indicate day or number of days that perceives pain in relation to your menses (can mark several options if considered necessary) do not answer this question if you do not suffer from Dysmenorrhea.

- 2-3-before menstruation
- The first day of menstruation
- Second or third day of menstruation
- No pain/ no dysmenorrhea

44. Do you feel that any of these symptoms: dysmenorrhea or other symptoms described in the previous question, limit you in their daily activities (going to class, attend practices, concentrate, and make sport...)?

- Yes
- No

45. Do you have children?

- Yes, for vaginal birth
- Yes, by caesarean section
- No
- Other:…..

46. Are you pregnant?

- Yes
- No

47. Do you use hormonal contraception?

- Yes
- No

48. Used analgesic to relieve menstrual symptoms

- Yes, self-medicates
- Yes, I so I have prescribed my healthcare professional
- No

49. If you take an analgesic during menstruation When do you take them?

- Days prior to menstruation
- During menstruation
- Only when the symptoms become worse

‘
